# The HOPE program: protocol for a stratified cluster randomized controlled trial to prevent psychoactive substance use and promote health in Moroccan middle school students

**DOI:** 10.1186/s13722-026-00691-1

**Published:** 2026-06-26

**Authors:** Hicham El Malki, Salma Ghofrane Moutawakkil, Abdelfettah El-Ammari, Mohammed El Amine Ragala, Fadoua Taoufik, Samir Elgnaoui, Khadija Zaidi, Imane Taoufiq, Said Chibri, Ghada Beniaich, Abdelghaffar El-Ammari, Hicham El Kazdouh, Achraf El Asri, Amine El Moutaouassil, Samira Abbouyi, Deyae Zerrouk, Kenza Kassab, Samira Bouazza, Karima El Rhazi, Btissame Zarrouq

**Affiliations:** 1 Laboratory of Epidemiology and Research in Health Sciences, Faculty of Medicine, Pharmacy, and Dental Medicine, Sidi Mohamed Ben Abdellah University, Fez, Morocco; 2 Higher Institute of Nursing Professions and Health Techniques of Fez, Regional Directorate of Health and Social Protection of the Fez-Meknes Region, Fez, Morocco; 3 Scientific Innovation in Sustainability, Environment, Education, and Health in the Era of Artificial Intelligence (Ecole Normale Supérieure), Sidi Mohamed Ben Abdellah University, Fez, Morocco; 4 Psychology Department, Private University of Fez, Fez, Morocco; 5 Addictology Center, Regional Directorate of Health and Social Protection of the Fez-Meknes Region, Fez, Morocco; 6 Psychology Department, Faculty of Letters and Human Sciences Sais, Sidi Mohamed Ben Abdellah University, Fez, Morocco; 7 Psychology Department, Faculty of Letters and Human Sciences Ain Chok, University of Hassan II Casablanca, Casablanca, Morocco; 8 Laboratory of Engineering, Electrochemistry, Modalization and Environment, Dhar Mehraz Faculty of Sciences, Sidi Mohamed Ben Abdellah University, Fez, Morocco; 9 National School of Public Health, Ministry of Health and Social Protection, Rabat, Morocco; 10 Regional Centre for Teaching and Training Professions, Tangier, Morocco

**Keywords:** Psychoactive substance, HOPE, School-based, Students, Psychosocial skills

## Abstract

**Background:**

Early adolescent psychoactive substance use poses major health and social risks. School-based programs enhancing psychosocial skills are effective for prevention. This study aims to develop, implement, and evaluate “HOPE”, a school-based program to prevent substance use and promote health among first-year middle school students in Fez, Morocco.

**Methods:**

A stratified cluster randomized controlled trial will be conducted across 12 middle schools, enrolling 1,000 first-year students (500 intervention, 500 control) and 60–90 parents. Stratified cluster randomization based on sociodemographic characteristics will ensure group balance. The intervention will be delivered over two years through 22 structured in-person group sessions (15 for students, 7 for parents) at participating schools. Data collection will involve structured student questionnaires and parent focus groups to assess a range of outcomes, including knowledge acquisition, attitude change, development of psychosocial skills, substance use patterns, and related behavioral and psychological effects. Measurable objectives include improvements in knowledge and psychosocial competencies, as well as reductions in substance use and associated harms. Expected outcomes include delayed initiation of substance use and enhanced student well-being.

**Discussion:**

We expect that the program will help to prevent and/or delay psychoactive substance use among first-year middle school students by reinforcing protective factors and reducing risk factors. We anticipate a reduction in current substance use, a decreased risk of abuse, and diminished physical and psychological effects. This study will evaluate the effectiveness of the "HOPE" program in reducing substance use and improving the physical and mental health of first-year middle school students in Fez. Its multi-year structure and potential positive outcomes could serve as a foundation for a national school-based prevention initiative in Morocco.

**Trial registration:**

Pan African Clinical Trial Registry PACTR202401711272288 (Registered on January 26, 2024).

## Introduction

### Background

The use of psychoactive substances (PAS), a multifaceted global issue, is currently acknowledged as a major societal problem [1, 2], with 284 million individuals aged 15–64 (5.6% of this demographic) reporting drug use in 2020 [3]. Early PAS initiation among adolescents represents a critical public health concern, with extensive research demonstrating both acute and chronic health consequences [4, 5] and consistently documenting elevated usage rates across diverse national contexts [6]. Moroccan epidemiological studies reveal particularly alarming trends, with school-based surveys showing early initiation patterns [7]. The third Global School-Based Student Health Survey (GSHS) of 2016 identified an increasing early prevalence of substance use among students aged 13 to 17 [8]. Similarly, the 2017 Mediterranean Survey on Alcohol and Other Drug Use in Schools (MedSPAD III) reported early initiation, with average ages of 15 for alcohol, 14.3 for tobacco, and 15.4 for cannabis [9]. Additionally, a cross-sectional study conducted in Morocco’s Centre-North region between 2012 and 2013 found that the average age of smoking initiation was 14, coinciding with the transition from middle to high school [7]. Among the 3,020 students surveyed, 16.1% reported smoking, with hookah (70.6%), snuff (42.8%), and chewing tobacco (35%) being prevalent [10]. Smoking rates were significantly higher in high school students (21.2%) compared to middle school students (11.9%) [10, 11].

Adolescent engagement in PAS use adversely affects psychosocial and neurocognitive development, increasing the risk of substance abuse, dependence, impaired cognition, aggression, social difficulties, and psychiatric disorders [12–16]. Preventing these effects is challenging, with the best strategy being to delay or prevent drug use onset [17, 18].

Evidence-based school prevention programs represent a validated strategy for delaying substance use initiation among adolescents. Their large-scale implementation offers an efficient.

public health approach to prevention [19, 20]. Multiple theoretically-grounded prevention models have been empirically developed, tested, and evaluated across different contexts and cultures [21–29]. Effective models typically integrate strategies focusing on knowledge, skills, and social influences [15, 23, 29, 30]. Among these models, empirical evidence suggests that the most effective programs are those that simultaneously combine the delivery of knowledge, the development of personal and social skills, and the targeting of social influence processes [31–34]. Prominent examples include *Life Skills Training* (LST), *Unplugged*, *Project ALERT*, *Lions Quest Skills for Adolescence*, and *Health4Life*, all of which have demonstrated significant effects in preventing substance use [28, 30, 35, 36]. These programs not only impart information about the nature and adverse consequences of substance use but also aim to develop psychosocial skills to help adolescents manage social pressures, resist substance offers, reduce motivations, and build resilience [23, 29, 33, 37]. Contemporary approaches increasingly emphasize the importance of incorporating the family environment by engaging parents in interventions [38–40].

Evidence-based school prevention programs draw upon established behavioral theories to explain behavioral change mechanisms. Social Learning Theory (SLT) [41] and Problem Behavior Theory (PBT) [42] describe observational learning and risk behavior development, while the Health Belief Model (HBM) [43] and Theory of Reasoned Action (TRA) and Theory of Planned Behavior (TPB) [44] explain decision-making processes. Social Norms Theory (SNT) [45] complements these by addressing substance use-related perceptual biases.

In the Moroccan context, the lack of research on prevention programs targeting PAS use through psychosocial skills development, coupled with the challenges of adapting existing models - often limited in accessibility and insufficiently aligned with the specific sociocultural needs of Moroccan adolescents - underscores the need for tailored interventions.

A stratified Cluster Randomized Controlled Trial (cRCT) will be conducted in Fez City to develop, implement, and evaluate the “HOPE” school-based prevention program, aiming to prevent PAS use and promote mental and physical health among Moroccan first-year middle school students through the development of psychosocial skills and parental involvement.

### Objectives

This study aims to:

1. Develop the “HOPE” program to prevent PAS use (tobacco, cannabis, alcohol, benzodiazepines, inhalants) by improving knowledge, attitudes, and psychosocial skills.
2. Implement and evaluate the program in six middle schools during the 2024-2025 and 2025-2026 school years, focusing on implementation aspects like dose, fidelity, and stakeholder feedback.
3. Assess the short-term impact on mediators such as knowledge, attitudes, and psychosocial skills in both intervention and control groups.
4. Evaluate the program’s effectiveness at 0, 6, 12, and 18 months on the age of initiation, substance use prevalence, and intention to use.
5. Examine the impact on physical symptoms (insomnia, violent behavior) and psychiatric symptoms (depression, anxiety, stress, suicidal risk) related to substance use.

## Materials and methods

### Reporting standards

This study protocol adheres to the SPIRIT 2025 statement and the TIDieR guidelines for intervention reporting.

### Study design

**Fig. 1:**
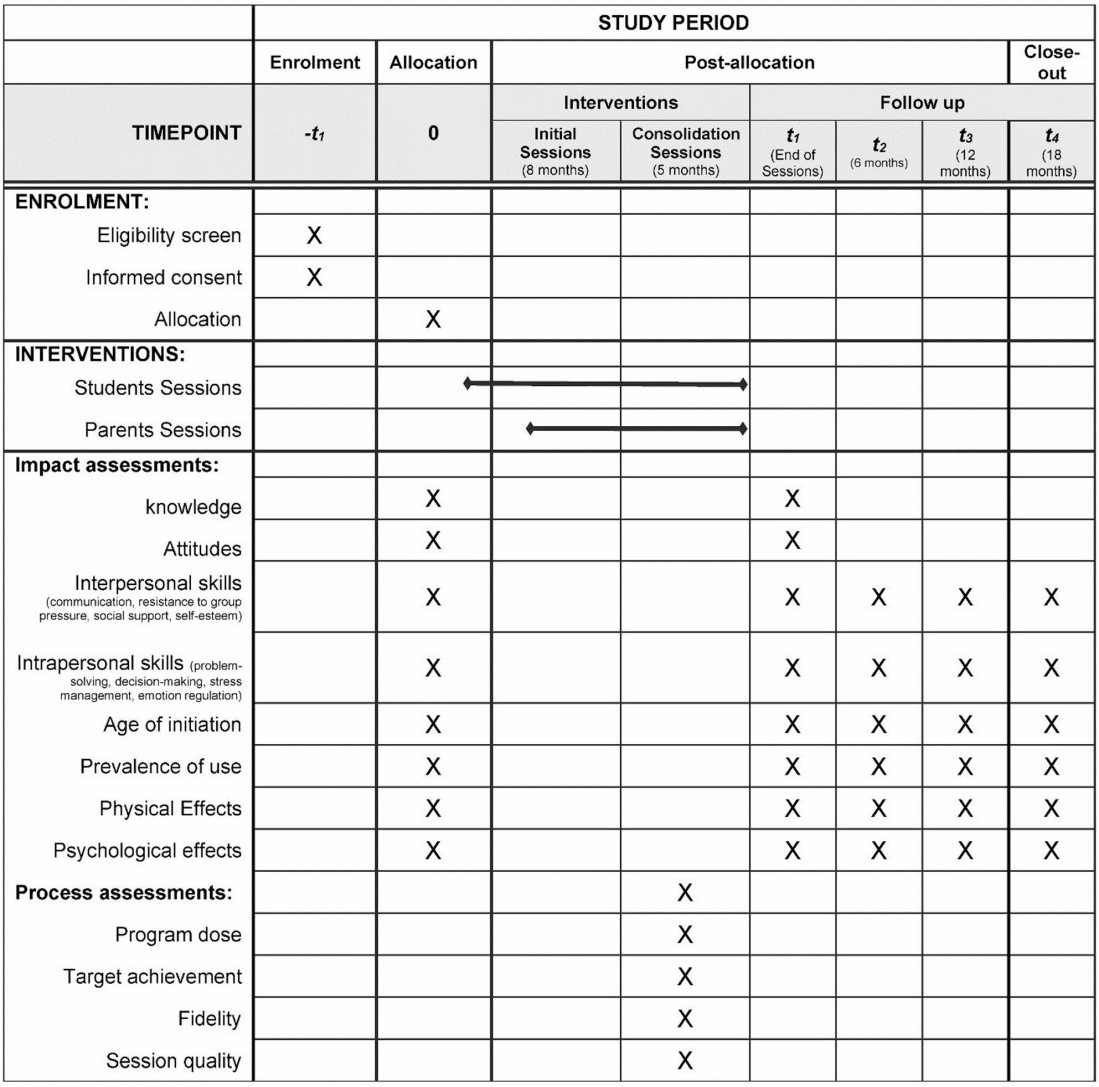
Standard protocol items: recommendations for interventional trials (SPIRIT); enrolment, interventions and assessments

A stratified cRCT will be conducted with first-year middle school students and their parents in Fez, spanning from 2024 to 2027. The research will involve 12 distinct middle schools and 1,000 students from 24 classes. Participants will be divided into an intervention group (n_IG_ = 500), receiving 15 program sessions over 13 months during the 2024–2025 and 2025–2026 school years, and a control group (n_CG_ = 500), which will be monitored without receiving any structured intervention to isolate the specific causal effect of the program by eliminating confounding factors, in accordance with the methodological standards of randomized controlled trials [46, 47]. Each group will be drawn from 6 separate schools (2 classes per school) to minimize contamination and prevent the Hawthorne effect [48]. Concurrently, 60–90 parents/guardians will participate in 7 parallel training sessions to support the intervention (Fig. 1).

### Study population

**Fig. 2:**
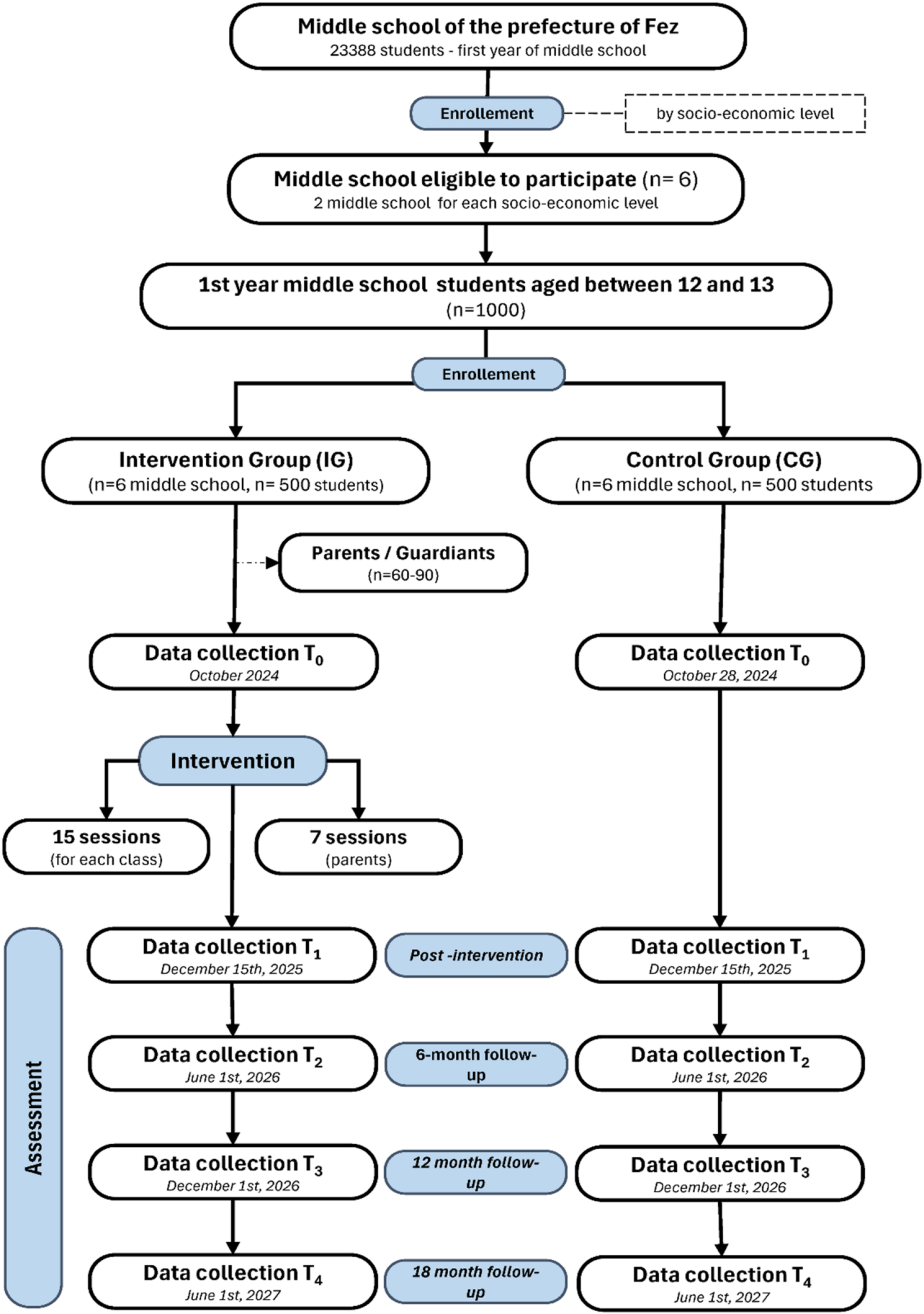
Flowchart of the overall design of “HOPE” program

First-year middle school students aged 12 to 13 at baseline, enrolled in public middle schools in the city of Fez, will take part in the study. This cohort was selected based on a combination of developmental, epidemiological, and methodological considerations: (i) they precede the average age of substance use initiation, which is approximately 14 years according to MedSPAD III [9], enabling timely prevention of risk behaviors; (ii) at this stage of development, consumption patterns are typically not yet established, providing an optimal window for intervention before experimentation begins; and (iii) the three-year middle school structure within the same institution facilitates stable longitudinal monitoring — unlike primary schools, where the transition to middle school complicates rigorous long-term follow-up. In addition, a subset of 10 to 20 parents or guardians of students from each intervention school will be invited to attend the training sessions and to qualitatively evaluate the “HOPE” program (Fig. 2).

#### Recruitment and informed consent

The Fez-Meknes Regional Academy of Education and Training has initiated the process of identifying middle schools that meet the eligibility criteria and will select 12 out of 40 public middle schools based on sociodemographic criteria (disadvantaged, intermediate, and well-off) to achieve a comprehensive representation of various backgrounds from which schools will be chosen. Six schools will implement the “HOPE” program, while the other six will serve as controls. After obtaining authorization, school heads will be informed via official letters and in-person meetings. Following confirmation of participation, students will be briefed through class meetings organized by the study team, outlining the study’s objectives and enrollment procedures.

In addition, printed consent packs will be sent to the students’ homes, comprising four key components: (1) an informative leaflet explaining the study and data collection procedures; (2) a written consent form for parents or guardians; (3) a written assent form for the students themselves; and (4) an additional written consent form for parents or guardians of students in the intervention group, granting them the option to attend the parent sessions if they choose to participate.

#### Eligibility criteria

Eligibility criteria required students to be 12 to 13 years old at the start of the study, currently enrolled in the first year of middle school, with both their own written consent and that of their parents or guardians. Parents were eligible if their children were included in the intervention group and provided written informed consent. Exclusion criteria included previous or current participation in another school-based PAS prevention program or refusal to attend all program sessions.

### Sample size

The sample size for this cluster-randomized trial was calculated through a two-stage process that integrated both conventional sampling theory and cluster-specific adjustments. Initial estimation followed standard simple random sampling methodology from a target population of 23,388 first-year middle school students across public schools in Fez, based on 2023–2024 statistics from the Ministry of National Education and Vocational Training, using the formula: n = [Z² × P(1-P)] / e² [49], where Z represents the 95% confidence interval critical value (1.96), ***P*** the expected proportion (set at 0.5 for maximal variance), and *e* the margin of error (3.2%). This calculation indicated a minimum requirement of 944 participants.

Given the hierarchical structure (students nested within schools) and the longitudinal nature of the study aiming to detect intervention-by-time interactions across four measurement waves (baseline plus three follow-ups), intra-cluster correlation (ICC) was accounted for using Heo and Leon’s [50], method specifically developed for longitudinal cluster randomized trials.

Twelve middle schools, with an average of 35 students per class, were selected for inclusion and equally allocated between intervention and control groups. Sample size calculations assumed a two-tailed significance level of α of 0.05, 80% statistical power, an ICC of 0.01 (based on educational intervention standards [51–54]), an average cluster size of 35 students per class, and a standardized minimum detectable effect size of d = 0.15, which aligns with findings from previous school-based prevention (reported effect sizes ranging from 0.15 to 0.30 [55–57]).

The cluster-adjusted calculation yielded a requirement of 914 participants. After incorporating an 8% attrition rate (derived from comparable school-based trials [56, 58–60]), the target increased to 987 students, which we rounded to 1,000 students (from 24 classes across 12 schools) for practical implementation.

The sample size calculation was primarily based on the expected intervention effects regarding proximal outcomes (knowledge, attitudes, and psychosocial skills). Distal outcomes of a clinical or behavioral nature, considered exploratory within the scope of the present study, were not incorporated into this power calculation.

For parents/guardians, a total of 60 to 90 will be recruited for training sessions and qualitative evaluations of the “HOPE” program. Based on qualitative study recommendations, which suggest a minimum of 50 participants for data saturation [61], participants will be selected based on availability and socioeconomic background: 20 to 30 from low-income, high-income, and middle-class neighborhoods each.

### Randomization

The randomization process will be organized into three distinct phases to ensure both a random and balanced allocation of schools and classes between the intervention and control groups.

Schools will initially be stratified into three sociodemographic categories (low, medium, affluent) based on predetermined criteria including geographic location, neighborhood income levels, and available institutional resources as determined by the Regional Academy of Education and Training. Within each stratum, four schools will be randomly selected using a computer-generated randomization sequence, with two assigned to the intervention group and two to the control group, yielding a total sample of twelve participating institutions equally divided between study conditions.

Following school selection, class-level randomization will be conducted within each participating institution through a sealed envelope procedure supervised by the Fez-Meknes Regional Academy to ensure procedural integrity. This stage will account for practical constraints including grade-level availability and scheduling considerations while preserving random allocation principles. Subsequently, a matching procedure will be implemented to enhance baseline equivalence between groups, focusing on key covariates including class size, gender distribution, and prior academic achievement as measured by standardized performance metrics from the preceding academic year.

The entire randomization procedure will be completed prior to the start of data collection and will be thoroughly documented to guarantee transparency, reproducibility, and methodological rigor.

### Intervention

The “HOPE” program is a Moroccan initiative aimed at preventing the use of PAS by reinforcing protective factors and reducing risk factors among middle school students. Its main objectives are to prevent or delay the initiation of PAS, to reduce consumption among current users, and to decrease the physical and psychological effects associated with PAS consumption.

#### Program theoretical basis

Any prevention program (or intervention) does not work by itself and is not what produces an effect. In fact, each intervention initiates a mechanism or a set of mechanisms that enable the production of specific effects. In addition, since every intervention is situated in a context, the interaction of the context and the intervention is what triggers—or not—the mechanism that leads to the effect or outcome. Several social psychology theories have been formulated and empirically tested in the context of diverse prevention programs to elucidate how the introduction of resources based on psychosocial skills within specific contexts can trigger mechanisms that generate positive results [62, 63].

**Fig. 3:**
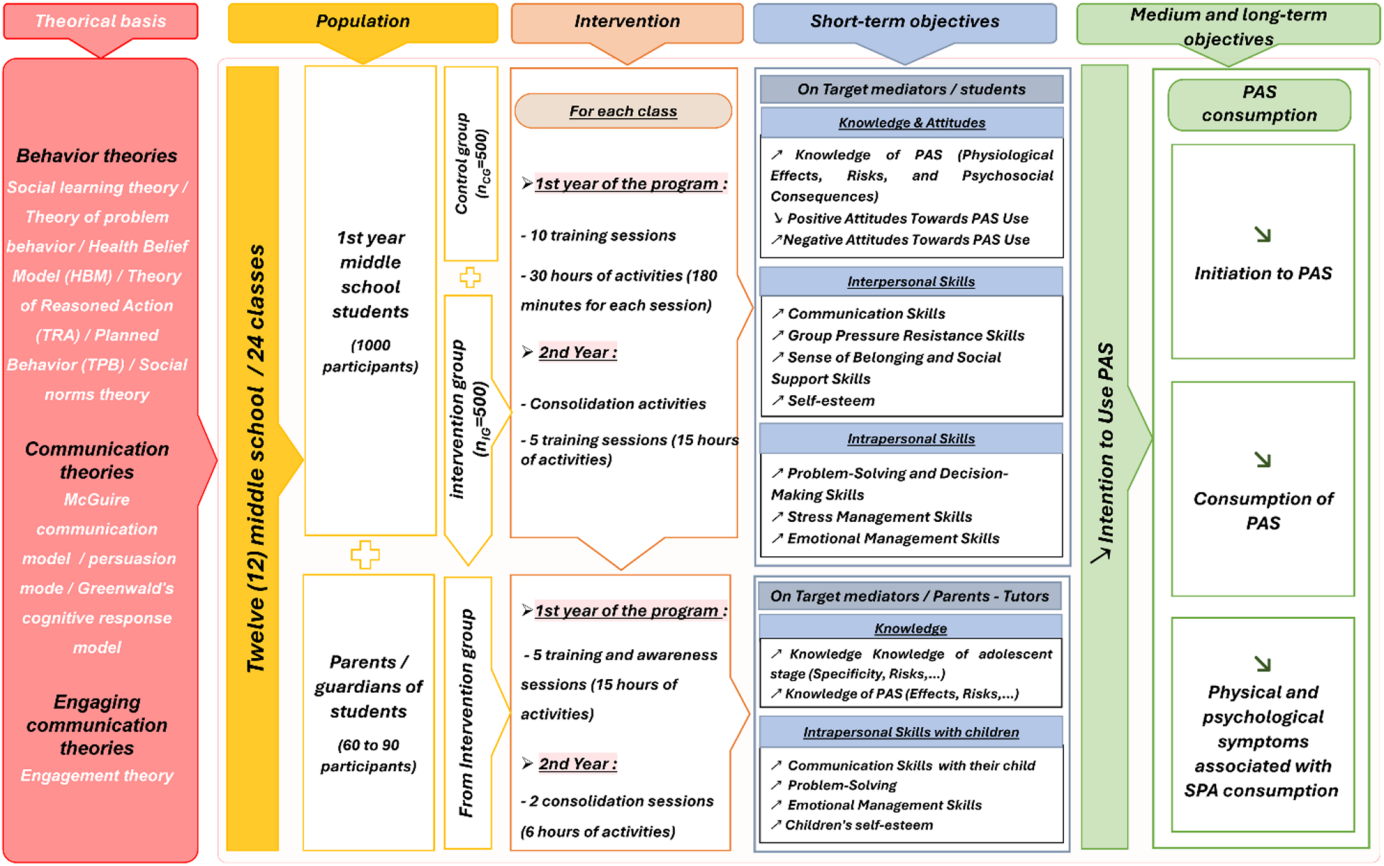
Theoretical framework of the “HOPE” program

The “HOPE” prevention program is being designed by incorporating several theories that will serve as the theoretical foundation for this research project: (i) behavior theories [social learning theory [64], the theory of problematic behavior [42], the health belief model (HBM) [65], the theory of reasoned action (TRA) and planned behavior (TPB) [44], and the theory of social norms [66], (ii) communication theories [McGuire communication model [67], the petty & Cacioppo persuasion model [68], Greenwald’s cognitive response model [69], and (iii) theory of engaging communication [engagement theory [70] (Fig. 3).

#### Causal pathways

The intervention is hypothesized to primarily influence primary outcomes directly targeted by the session content, including adolescents’ knowledge, attitudes, and psychosocial skills. These direct effects are grounded in the theoretical frameworks underpinning the intervention: behavioral theories explain changes in knowledge, attitudes, and skills, while communication and engagement theories elucidate persuasion and active participation.

Changes in these variables are conceptualized as mediating mechanisms linking the intervention to behavioral outcomes. Strengthening psychosocial skills and adjusting perceived norms reduce vulnerability to peer influence, strengthen intentions not to use PAS, and decrease the likelihood of initiation or continued use. Consequently, behavioral indicators (prevalence, age of initiation, patterns of use) are considered secondary outcomes, reflecting downstream effects of changes in cognitive and psychosocial processes.

Finally, mental health indicators (anxiety, depression, stress, sleep disturbances) are distal exploratory outcomes, expected to change indirectly through reduced risk behaviors and improved coping.

#### Sessions

The program will consist of a total of 22 training sessions, with 15 sessions dedicated to students in the intervention group, and 7 sessions for their parents. Within the student sessions, during the first school year of program implementation (2024–2025), 10 sessions will be scheduled with the aim of raising knowledge of the risks associated with PAS, challenging the normalization of PAS use by evaluating attitudes toward acceptability and negative consequences, and fostering psychosocial skills, in particular interpersonal (communication, group dynamics, social support, and peer influence/pressure), as well as intrapersonal skills (self-esteem, problem-solving, decision-making, stress and emotion management) *(*Table 1*)*. 5 additional consolidation sessions will be provided in the second school year (2025–2026) according to the needs identified at the end of the first phase, with the aim of strengthening and consolidating the knowledge and skills acquired in the first year.

The design of the sessions prioritizes alignment with the developmental stage of the Moroccan adolescents involved, with content, pedagogical methods, and communication strategies adapted to their cognitive maturity and learning styles, in order to optimize comprehension, engagement, and overall intervention effectiveness.

For parents, of the 7 sessions planned, 5 will be delivered in the first school year of the program and 2 in the second year. These initial 5 sessions are designed to empower parents with essential skills to navigate the complexities of raising adolescents effectively and fostering positive family dynamics. The focus will be on enhancing their understanding of adolescence as a critical and challenging life stage, gaining insights into drug addiction (its various manifestations and risks), and acquiring effective communication techniques to facilitate improved parent child relationships, and bolster the psychosocial abilities of their adolescents (Table 2).

Parental involvement is a foundational element of the intervention, serving to: (a) enhance parents’ ability to provide educational and preventive support; (b) foster a family environment that ensures alignment between the program’s objectives and the home setting; and (c) contribute to the development of adolescents’ psychosocial skills, while also engaging parents in the program’s evaluation process.

All the sessions planned as part of the program will take place in person at the various middle schools concerned. Each session will be led by a speaker specializing in the area covered by the session objective and will last 3 h. This time volume will be spread over two sequences for each session, for both students and parents, in order to allow sufficient time for the development of each of the desired skills [71]. The sessions will be scheduled according to the duration of each stage of the program, at the rate of two sessions per week for students (considering class schedules), and one session per week for parents/guardians.

The team designated to conduct the sessions will include a total of 13 individuals: 10 facilitators will be responsible for students’ sessions, and 3 for parent sessions. Health professionals (doctors, psychotherapists, nurses, social assistants), teachers, university professors, and members of associations working in the field of risk reduction linked to PAS, will deliver the various sessions of the “HOPE” program. These profiles are selected in accordance with the recommendations of the United Nations Office on Drugs and Crime (UNODC) and the World Health Organization (WHO) for delivering this type of intervention [72, 73].

**Table 1:** Overview of the program plan (student sessions)

Lesson	Title	Time (mn)		Aim	Resources, materials
		Sequence 1	Sequence 2		
1	*Opening of the Program “HOPE”*	90	90	Introduce the program and the various sessions to be carried out. Determine the program’s objectives in the short, medium and long term. Specify the rules to be adopted and followed during the sessions.	Poster Program booklet Class charter
2	*Risky behaviors*	90	90	Understanding the concept of risk behavior. Differentiate between risk behavior and healthy behavior. understand the relationship between risk behavior and psychoactive substances.	Presentation Worksheets for group work Game
3	*Drugs: false representations*	90	90	Correct the information concerning the PAS. Reflect on the differences between personal opinion and reality. Reassess students’ norms about PAS.	Presentation Question sheets Plenary discussion
4	*Communication*	90	90	Knowing and identifying existing communication styles. Distinguish between verbal and nonverbal communication. Understanding the importance of good communication.	Situation play Situation sheets Group work
5	*Emotions and stress management*	90	90	Learning to describe and differentiate between emotions. Reflect and express emotions appropriately. Learn to relax in stressful situations.	video presentation Role play Relaxation session
6	*Critical/creative thinking*	90	90	learn to evaluate information critically. Improve decision-making based on reliable data. Encouraging creative thinking and self-control.	Presentation Situation sheets Group work
7	*Group membership /peer pressure*	90	90	Identify situations where pressure is being exerted on a person. Learn to stand up for your rights and say no. Developing respect for the rights and opinions of others.	Video presentation Plenary discussion Game
8	*Resolving problems*	90	90	Analyze a problem situation and provide a solution. Establish a procedure to choose the right response to the problem. Working as part of a team.	Situation sheets Role play Plenary discussion
9	*Self-confidence*	90	90	Encouraging self-awareness and knowledge of others. Encourage the identification of positive qualities in oneself and in others. Improve self-esteem.	Work in pairs Situation play Plenary discussion
10	*Decision-making*	90	90	Analyze a problem situation and provide a solution. Establish a procedure to choose the right response to the problem. Collaborating as a team to make the right decision.	Situation sheets Game group work

**Table 2:** Overview of the program plan (parents/guardian’s sessions)

Lesson	Session title	Time (mn)		Aim	Resources, materials
		Sequence 1	Sequence 2		
1	*Opening of the Program “HOPE”*	90	90	Introduce the program and the various sessions to be carried out. Specify the program’s objectives in the short, medium and long term.	Poster Program booklet
2	*Adolescence: what parents need to understand*	90	90	Understanding the adolescent stage and its needs. Demonstrate empathy toward children to manage family conflicts. as effectively as possible. Be familiar with educational models and their characteristics.	Presentation Plenary discussion
3	*Addiction: what parents need to know*	90	90	Information on PAS. Generate a more realistic view of PAS addiction. Correct the information about drugs. Re-evaluate parents’ norms about PAS. Know the risk and protective factors that encourage young to take drugs.	Brainstorming Question sheets Presentation Plenary discussion
4	*Successful communication*	90	90	Be aware of the importance of talking to your child. Talking to your child about the dangers of using PAS Establish rules and limits regarding PAS	Video Situation play Worksheets for group work
5	*Parents and children’s psychosocial skills*	90	90	Developing your child’s self-esteem Learn to be assertive in managing day-to-day problems with your children. Obtain strategies for dealing with adolescent problems.	Presentation Role play Plenary discussion

#### Implementation

The implementation of the “HOPE” program will be carried out in four phases. Initially, agreements and policies were established with the academy and provincial directorate of education levels. These agreements and policies will facilitate the smooth implementation of the program in state middle schools.

In the second phase (September - December 2023), comprehensive training sessions for the trainers responsible for implementing the program in the selected schools are organized by the program designers in collaboration with the Fez-Meknes Regional Health Authority and Sidi Mohamed Ben Abdellah University. These sessions aimed to ensure program fidelity by equipping facilitators with essential implementation knowledge and skills necessary for the effective execution of the program. A multidisciplinary team of experts—including school psychologists, addiction specialists, health promotion educators, and certified psychosocial education trainers—delivered twelve hours of training across six modules.

The curriculum addressed five core domains: adolescent developmental psychology, clinical and psychosocial dimensions of addiction, evidence-based prevention strategies, psychosocial skill development techniques, and interactive pedagogical methods. Training employed an experiential learning approach through theoretical instruction, case study analyses, role-playing exercises, program session simulations, and collaborative group work.

The training culminated in participant-led demonstrations evaluated by a steering committee comprising psychologists, sociologists, epidemiologists, and psychiatrists. This evaluative component verified content mastery and alignment with predefined pedagogical objectives while ensuring implementation quality through expert feedback.

To enhance adherence among participants (students and parents) to the program sessions, the training provided to trainers emphasized strategies for personalized support, ensured clear and consistent communication regarding the program’s benefits, incorporated regular reminders and feedback mechanisms, and focused on identifying and addressing individual barriers to participation.

The third phase will entail the selection of middle schools that will participate in the program. A careful selection process will be conducted to ensure that the chosen schools align with the program’s objectives and requirements.

Finally, the fourth phase will involve the actual implementation of the “HOPE” program, along with an evaluation process. The evaluation will assess the program’s effectiveness and identify areas that can be improved. Based on the evaluation results, adjustments and enhancements will be made to strengthen the program’s impact during the second year of implementation (consolidation phase).

#### Program timeline

The sessions of the “HOPE” program will be scheduled over a span of two years, covering the school years 2024–2025 and 2025–2026. The initial 10 student sessions will occur from November 1st, 2024, to June 30th, 2025, followed by 5 consolidation sessions from October 15th to December 15th, 2025. Concurrently, 7 parent sessions will be conducted: 5 sessions from February 1st to June 30th, 2025, and 2 consolidation sessions from October 15th to November 30th, 2025.

### Assessment

#### Impact assessment

A mixed methods process evaluation of the impact of the “HOPE” program will be conducted: ***-****Quantitative data analysis*: A quantitative evaluation of the program’s impact, encompassing the following stages: (1) Baseline measurement (T_0_) will take place on October 28, 2024 (prior to the intervention): Assess PAS-related knowledge, attitudes, substance use prevalence, psychological and physical symptoms, and psychosocial skills among both intervention and control groups. (2) Post-intervention measurement (T_1_) on December 15th, 2025: Evaluate changes in knowledge, attitudes, psychosocial skills, substance use prevalence, and related symptoms, comparing intervention and control groups. (3) Short-term follow-up (T_2_) on June 1st, 2026 (6 months post-intervention). (4) Middle-term follow-up (T_3_) on December 1st, 2026 (12 months post-intervention). (5) Long-term follow-up (T_4_) on June 1st, 2027 (18 months post-intervention).

*- Qualitative data analysis*: Focus groups with parents of students will be conducted following the training sessions (T_1_) to evaluate the “HOPE” program’s effectiveness and its broader impact on drug use among students (Table 3).

Immediate post-intervention assessment (T_1_) is primarily intended to capture proximal changes, whereas follow-up assessments (T_2_–T_4_) are designed to evaluate delayed behavioral and clinical effects.

#### Process assessment

In addition to assessing the program’s impact, a process evaluation will monitor its implementation to ensure alignment with the set objectives. Key indicators will include program dose (session completion), target achievement (student/parent participation), fidelity (materials, duration, content), and session quality (speaker, climate, strengths, weaknesses). Selected teachers associated with the middle schools where the program is being implemented will assess dose, target achievement, and fidelity, while both teachers and students will evaluate session quality. All indicators will be assessed quantitatively and qualitatively at the end of each session and at the program’s conclusion (Table 3).

**Table 3:** Data collection techniques, participants, time, instruments, and variables used in the process evaluation of the HOPE Program

Type	Technique	Participants	Timing	Instrument	Variables
Quantitative	*Diagnostic assessment*	Students: Intervention group + control group (*n* *= 1000)*	T_0_ Pre-intervention	Self-report questionnaire	Knowledge, attitudes, use of PAS, physical and psychological state and psycho-social skills
	*Intervention assessment*	Students: Intervention group (*n* *= 500)*	T_1_ At the end of the 15 HOPE sessions	Self-report questionnaire	Knowledge, attitudes, skills, consumption, and psycho-social skills.
		Students: Intervention group + control group (*n* *= 1000)*	T_2_ 6-month follow-up	Self-report questionnaire	Consumption of PAS, age of initiation, physical and psychological symptoms
		Students: Intervention group + control group (*n* *= 1000)*	T_3_ 12-month follow-up	Self-report questionnaire	Consumption of PAS, age of initiation, physical and psychological symptoms
		Students: Intervention group + control group (*n* *= 1000)*	T_4_ 18 months follow up	Self-report questionnaire	Consumption of PAS, age of initiation, physical and psychological symptoms
	*Process assessment*	Students: Intervention group (*n* *= 500)* + Teachers	AFTER every session	Self-report questionnaire	Dose, fidelity, target achievement, and sessions
Qualitative	*Focus Group* (*n* *= 60–90* *participants)*	Parents of students of intervention group	T_1_ At the end of the 7 sessions for parents	Focus Group	Consumption of PAS, physical and psychological symptoms present in their children’s
	*Process assessment*	Teachers	At the END of the HOPE sessions	Focus Group	Sessions & Program

### Outcomes

Expected outcomes are categorized into primary, secondary, and exploratory domains, in line with the hypothesized causal pathways.

#### Primary outcomes

include PAS consumption metrics, such as age of initiation, prevalence of use (tobacco, cannabis, alcohol, benzodiazepines, inhalants), and related behavioral issues.

#### Secondary outcomes

encompass knowledge of PAS (physiological effects, risks, psycho-social consequences) and attitudes toward PAS (negative/positive). Interpersonal skills (communication, resistance to group pressure, social support, self-esteem) and intrapersonal skills (problem-solving, decision-making, stress management, emotion regulation) will also be evaluated.

#### Exploratory outcomes

comprise the physical and psychological effects associated with PAS use, including sleep disturbances, aggression, depression, anxiety, stress, and suicide risk.

As long-term outcomes, these measures are unlikely to change measurably in the immediate post-intervention period. Interpretation will therefore center on follow-up assessments, with early results remaining exploratory.

### Data collection

#### Quantitative data

For impact program evaluation, data collection will involve a self-administered questionnaire written in Arabic, customized to suit the Moroccan context, student vocabulary, and the local language associated with various PAS. This questionnaire, developed by our project team and inspired by internationally used surveys, will include: (1) sociodemographic information, including age, gender, socioeconomic status, place of residence, hobbies, parental status, and educational level; (2) family environment, exploring aspects like parents’ marital status, family structure, relationships with family members, and consumption patterns in the family context; (3) school environment, assessing students interactions with school, classmates, teachers, and academic performance indicators such as grades, absenteeism, and running away; (4) friendship environment, investigating peer associations, relationships, and friends’ influence; (5) knowledge, opinions, and attitudes toward psychoactive substances; (6) substance use, covering initiation age, prevalence, and behavioral issues, with the utilization of validated psychometric scales like: Car, Relax, Alone, Forget, Friends, Trouble (CRAFFT) - (χ²/df = 1.91, Comparative Fit Index (CFI) = 0.98, Normed Fit Index (NFI) = 0.97, Root Mean Square Error of Approximation (RMSEA) = 0.05, SRMR = 0.03) [74], Cannabis Abuse Screening Test (CAST) (χ2/df = 2.23, RMSEA = 0.07, SRMR = 0.02, CFI = 0.99, NFI = 0.98) [75], and Hooked on Nicotine Checklist (HONK) - (χ²/df = 3.31, CFI = 0.98, Tucker-Lewis Index (TLI) = 0.96, RMSEA = 0.09, SRMR = 0.06) [76]; (7) physical health, incorporating insomnia assessment using the Insomnia Severity Index (ISI) scale - (Cronbach’s α = 0.83, Concordance Correlation Coefficient (CCI) = 0.99, kappa coefficient = 0.76) [77], and questions about violent behavior; (8) mental health, exploring potential traumatic experiences, depression, anxiety, stress and suicidal risk by employing scales like Depression, Anxiety and Stress Scale – 21 Items (DASS-21) - (Cronbach’s α = 0.88, CFI = 0.97, TLI = 0.95, RMSEA = 0.03) [78], and suicidal risk in International Neuropsychiatric Interview (MINI) - (Cronbach’s α = 0.89, sensitivity = 0.82, specificity = 1.00, Positive Predictive Value (PPV) = 1.00, Negative Predictive Value (NPV) = 0.98) [79]; and (9) psychosocial skills, evaluating social, cognitive, and emotional skills, including communication, empathy, resistance, negotiation, cooperation, advocacy, decision-making, critical thinking, self-assessment, emotion regulation, stress management, self-evaluation, and self-esteem.

Data will be collected using tablets provided as part of the program, enabling efficient data capture, reducing questionnaire completion time, and minimizing respondent burden and fatigue.

For process evaluation, data will be collected from the teachers of the establishments through a self-administered questionnaire at the end of each session. This questionnaire, consisting of questions with yes/no response options, will assess the delivery of the session, the number of students/parents present, materials used, session duration, content, speaker, climate, and strengths and weaknesses observed. Moreover, students will be provided with a self-administered questionnaire that exclusively includes the same set of questions for session evaluation (covering the speaker, the session atmosphere, and the strengths and weaknesses observed during the session).

#### Qualitative data

Qualitative data will be collected at two levels during the intervention: - for the evaluation of the impact of the “HOPE” program and the phenomenon of PAS use among adolescents with parents, and - to complete the evaluation of the program process at the end of the intervention with teachers from the participating schools. These data will be collected using the focus group technique which constitutes a form of small group interview which will aim to explore everything related to the subject of the study.

Consistent with the program’s objectives, the key domains explored in the interview guides will include perceived behavioral and psychosocial changes in adolescents related to substance use, parent–child communication, family dynamics and environment, parents’ perceptions of the HOPE program, and barriers and facilitators encountered during program implementation.

The discussions in each focus group will be led by a moderator and an observer, both of whom will be trained to perform this task. The moderator’s role will be to facilitate verbal communication with the participants, while the observer’s role will be to note the various forms of nonverbal communication such as gestures, mimics, and silence that he or she felt would be useful in enriching and complementing the verbal communication. The discussions will be conducted in the Moroccan dialect. The focus group interviews will be audio recorded after obtaining permission from the participants.

To encourage discussion among parents, and since the subject of PAS use is a taboo and sensitive topic in the Moroccan context, focus groups will be conducted with fathers and mothers separately.

The total number of focus groups will not be fixed in advance. Given the focused aim of the study and the specificity of the participant groups, sample size will be guided by the principle of information power, whereby a smaller sample is sufficient when the data collected are specific and directly relevant to the research question [80]. Data collection will proceed iteratively with concurrent analysis until thematic saturation is reached [81]. Saturation will be considered achieved when no new codes emerge from two consecutive focus groups within the same participant category (fathers, mothers, or teachers), and when the research team judges that the data provide a sufficiently rich account to address the research questions.

Data collection and analysis will be conducted iteratively, allowing insights from early focus groups to inform subsequent data collection and support the refinement of emerging themes.

### Statistical methods

Statistical analysis of the quantitative data will be conducted using the **R** programming language. Program effectiveness will be operationalized as between-group differences in the trajectories of primary and secondary outcomes over time, estimated using linear mixed-effects models for repeated measures [82]. Models will include fixed effects for group (intervention vs. control), time (T_0_ - T_4_, modeled as a categorical variable to allow for non-linear changes over time), and their interaction, along with relevant covariates identified a priori.

The primary parameter of interest will be the group-by-time interaction term, assessing whether outcome trajectories differ significantly between the intervention and control groups over the study period, with statistical significance evaluated using likelihood ratio tests (LRT) at a two-sided α = 0.05 level [83]. Random intercepts will be included to account for within-subject correlation arising from repeated measures and for the hierarchical structure of the data (students nested within classrooms/schools) [84].

The models will explicitly account for the hierarchical structure of the data, characterized by repeated measures within each student and the clustering of students within classrooms and schools [85]. Specifically, repeated measurements (Level 1) will be taken from students (Level 2), who are nested within classrooms (Level 3), which are in turn nested within schools (Level 4) [86]. Accordingly, random intercepts will be specified at the student, classroom, and school levels to account for within-group correlation. Random slopes for time may also be included, if supported by model fit and convergence, to capture individual variability in outcome trajectories.

Descriptive analyses (means, standard deviations, and proportions) will be performed to summarize baseline characteristics of the study population. Bivariate analyses (proportions, means), and multivariate analyses (logistic and linear regression models) will be carried out to investigate potential associations between independent variables, including the prevalence of PAS use and various aspects of mental and physical health - such as the prevalence of PAS use and various mental and physical health indicators (depression, anxiety, stress, suicidal risk, insomnia, and violent behaviors) - and dependent variables encompassing sociodemographic, academic, and family factors.

Variables associated with the outcome at *p* < 0.20 in bivariate analyses will be considered for inclusion in the multivariable models during a preliminary selection step. However, final selection will not rely exclusively on this statistical criterion but will also incorporate theoretical and conceptual considerations drawn from the existing literature and the study framework.

Key covariates (socioeconomic status, parental education, family structure, peer influence, and parental substance use) will be defined a priori and retained regardless of their significance in bivariate analyses [87–89]. All selected covariates will be integrated into the mixed-effects models as fixed effects, allowing assessment of their relationships with outcomes over repeated measurements while adequately accounting for the hierarchical structure of the data.

Multivariate analyses, including logistic and linear regression models, will be performed to identify independent factors associated with the outcomes. Results will be reported as odds ratios or regression coefficients with corresponding 95% confidence intervals, and statistical significance will be set at *p* < 0.05. Potential confounding factors, including socioeconomic status, peer influence, and parental substance use, will be assessed at baseline and adjusted for as covariates in the mixed-effects models to minimize confounding bias.

In the event of missing data, appropriate and robust statistical methods will be employed, including multiple imputations and mixed-effects models, suitable for repeated measures and capable of handling incomplete data under the Missing At Random (MAR) assumption. Patterns of missingness will be explored, and sensitivity analyses will be performed if necessary to assess their influence on the study outcomes.

For qualitative data, all audio recordings obtained from the data collection phase will be organized into two distinct corpora: one comprising teacher handouts and another comprising parental responses. Observers’ notes on nonverbal communication will be integrated into the corresponding corpus. All audio recordings will be transcribed verbatim and, where necessary, translated into the language of analysis.

Textual data will be manually and independently analyzed by two coders using Braun and Clarke’s method of thematic analysis [81]. This approach involves identifying, analyzing, and reporting patterns (themes) within qualitative data in relation to the research question. The analysis will follow the six phases outlined by Braun and Clarke: familiarization with the data, generation of initial codes, search for themes, review of themes, definition and naming of themes, and production of the final report [81].

To ensure analytic rigor, several strategies will be employed throughout the qualitative analysis. Coding will be primarily inductive, allowing themes to emerge from the data, while remaining informed by the interview guide domains and the program’s theoretical framework. Two researchers trained in qualitative analysis will independently analyze all transcripts. Prior to formal coding, they will familiarize themselves with the data and establish a shared preliminary coding framework. Discrepancies in coding or interpretation will be discussed until consensus is reached. Should disagreement persist, a third researcher will be consulted. Final themes will be reviewed by the broader research team to ensure interpretive coherence and fidelity to the data. Throughout the process, researchers will document their assumptions and potential biases in a reflexive manner.

An audit trail will be maintained to document analytic decisions and the steps of theme development, thereby enhancing the transparency and dependability of the analysis.

### Data monitoring

Data obtained during the study will be treated with strict confidentiality, and only authorized members of the research team will be granted access to the questionnaires and focus group materials. All consent forms, questionnaires, and focus group recordings will be securely stored within the premises of the responsible researcher for the entire duration of the study and will be securely disposed of two years after the study’s completion. To maintain the anonymity of participants during the monitoring and evaluation processes, unique codes will be assigned to the questionnaires and focus group data instead of using participants’ names.

## Discussion

This study protocol presents a cRCT designed to evaluate the impact of the “HOPE” School-Based prevention program on PAS consumption and its associated psychological and physical symptoms among Moroccan students. The primary objective of this research is to gauge the effectiveness of the intervention. Additionally, the research will collect data on various aspects, including knowledge, attitudes, and psychosocial skills, which may play a role in mediating the program’s effectiveness. The results may provide empirical support for integrating evidence-based prevention strategies into national educational policies, potentially informing the development of a standardized, competency-focused intervention framework for adolescent substance use prevention in Morocco.

### Strengths and limitations

One significant strength of this study is its development of a school-based prevention program against psychoactive substances, tailored by Moroccan experts to fit the specific needs of the Moroccan context, especially among adolescents. This adaptation draws from successful approaches used in similar contexts. The program will not solely focus on enhancing students’ personal and social skills, it will also extend its reach to address the family context by involving parents and guardians in training sessions regarding PAS and fostering safe behaviors with adolescents.

Furthermore, the study’s thoroughness is evident in its evaluation, which examines both the immediate and long-term effects of the “HOPE” program. With follow-up measurements at 6, 12 and 18 months, researchers can gain insights into how the program impacts participants in the short, medium, and long term.

Another important aspect is the study’s commitment to including a representative sample of 1000 students from the public schools in the Fez Prefecture. This diverse sample is carefully selected by a simple random process of both schools and classes for both the intervention and control groups.

In terms of limitations, the main constraint will be related to the potential high attrition rates of student over the three-year follow-up period, which could introduce bias into the study, although it is worth noting that this challenge is commonly encountered in longitudinal research [90, 91], and the goal is to try to mitigate it by increasing the theoretical sample size (an additional 60 students will be recruited to account for this), and by ensuring a thorough explanation of the importance of the intervention to both students and their parents/guardians. Additionally, the program sessions will be carefully planned to avoid imposing a burden on the students.

An additional limitation of the study is that the data collected at different points in the survey will be based on student and parent self-reports. Although this method is subject to potential measurement biases, including social desirability bias and recall bias, it remains the most recommended approach for assessing behaviors in adolescents and has demonstrated high validity in studies examining drug-related behaviors [15, 27, 92, 93]. Furthermore, it represents the only valid alternative since biological measurements would not be suitable for such a large sample and at the early stages of substance consumption [94, 95]. Strategies such as using validated anonymous questionnaires and conducting sensitivity analyses have been planned to minimize and assess the impact of these biases.

Moreover, the absence of an active control group may introduce potential placebo or expectancy effects, which could influence the comparison of outcomes in this study. However, several measures will be implemented to minimize these risks and maintain scientific rigor, including a strictly parallel evaluation timeline, blinded outcome assessors, the use of validated standardized questionnaires, and covariate control through multivariate analysis. This approach is consistent with pioneering school-based prevention trials [23, 96], where the absence of an active intervention in the control group nevertheless allowed for the demonstration of clear preventive effects.

Program implementation may face several obstacles, including disparities in resources between schools, challenges in sustaining long-term institutional engagement, low student participation rates, and scheduling conflicts with academic calendars.

Moreover, the multi-year design and intensity of the intervention represent both a methodological strength and a potential limitation. While enabling the progressive reinforcement of psychosocial skills and the adoption of protective behaviors toward psychoactive substance use, they may also constrain program sustainability, particularly in resource-limited educational settings, and could hinder broader scalability. All of these challenges will be systematically monitored through process evaluation metrics to guide program adaptation and enhance future scalability.

Finally, another important limitation concerns the logistical and contextual constraints that influenced the choice of the number of participating parents. These include the limited availability of qualified facilitators, who are few and highly specialized in this type of intervention in our context; scheduling conflicts due to the academic calendar, which prevent the delivery of an equivalent number of sessions to both students and parents without delaying the overall implementation and follow-up process; historically low parental engagement in such school-based programs, often due to confidentiality concerns or a lack of perceived relevance; and budgetary constraints that limit the logistical resources required to involve a larger parent sample.

## Conclusion

This study will design and assess a school-based prevention program targeting PAS consumption, as well as some aspects of the physical and mental health of adolescents in the middle schools of Fez city in Morocco. The outcomes of this study will provide insights into the effectiveness of the “HOPE” prevention program, with the aim of its potential expansion at national level.

## Data Availability

No datasets were generated or analyzed during the preparation of this research protocol. Upon completion of the study, all resulting data will be deposited in an open access public repository, and a permanent URL/DOI link will be provided at that time.

## Abbreviations

CAST: Cannabis Abuse Screening Test
CG: Control Group
CRAFFT: Car, Relax, Alone, Forget, Friends, Trouble
DASS-21: Depression, Anxiety and Stress Scale – 21 Items
GSHS: Global School-based Student Health Survey
HBM: Health Belief Model
HONC: Hooked on Nicotine Checklist
ICC: Intra-cluster Correlation
IG: Intervention Group
ISI: Insomnia Severity Index
LRT: Likelihood Ratio Tests
MAR: Missing At Random
MedSPAD: Mediterranean School Survey Project on Alcohol and other Drugs
M.I.N.I.: Mini-International Neuropsychiatric Interview
NFI: Normed Fit Index
NPV: Negative Predictive Value
PBT: Problem Behavior Theory
PPV: Positive Predictive Value
RMSEA: Root Mean Square Error of Approximation
SLT: Social Learning Theory
SNT: Social Norms Theory
SRMR: Standardized Root Mean Square Residual
cRCT: Stratified Cluster Randomized Controlled Trial
TLI: Tucker–Lewis Index
TPB: Theory of Planned Behavior
TRA: Theory of Reasoned Action
UNODC: United Nations Office on Drugs and Crime
WHO: World Health Organization
